# Visceral Kaposi’s Sarcoma as a Presentation in a Newly Diagnosed HIV-Infected Man: A Case Report

**DOI:** 10.7759/cureus.23339

**Published:** 2022-03-20

**Authors:** Zeinab El Mawla, Hiba Ghannoum, Michelle Saliba, Afaf Michel Minari, Hassan M Kanaan

**Affiliations:** 1 Internal Medicine, Lebanese University Faculty of Medical Sciences, Beirut, LBN; 2 Oncology, Lebanese University Faculty of Medical Sciences, Beirut, LBN; 3 Infectious Disease, Rafik Hariri University Hospital, Baabda, LBN; 4 Hematology and Medical Oncology, Military Hospital, Beirut, LBN

**Keywords:** c-art, hiv, visceral, sarcoma, kaposi

## Abstract

Kaposi’s sarcoma is an angioproliferative malignancy due to human herpesvirus-8 and is associated with immunosuppression. Although most cases are cutaneous and resolve with treatment of the underlying condition, few cases present with organ involvement and have a fulminant course. We present a case of a 24-year-old sexually active man who presented with fulminant visceral Kaposi’s sarcoma, without cutaneous involvement. He presented with anasarca, high fever, hypoalbuminemia, and anemia on day five of antiretroviral therapy (ART). There was clinical improvement after the first dose of liposomal doxorubicin. However, given that he developed refractory pancytopenia, with disease relapse by the third week, he received a second dose of doxorubicin, with no clinical improvement, and the patient died with multi-organ dysfunction on day 22 of presentation. The main treatment is liposomal doxorubicin with ART, and the disease is typically associated with a poor prognosis.

## Introduction

Kaposi's sarcoma (KS) is an angioproliferative tumor associated with the presence of herpesvirus-8 (HHV-8) in more than 90% of the lesions. The mode(s) of transmission of HHV-8 remains unclear, but epidemiologic and virologic data suggest that saliva is a source of the infectious virus and may be an important route of transmission as well as sexual contact [1]. KS was first described in 1872 by Moritz Kaposi, an Austro-Hungarian dermatologist, in five patients with multifocal disease [2]. KS is a malignancy of the lymphatic endothelial cells that is often highly aggressive in people with HIV and severe immunodeficiency. In KS, the majority of lesions begin on the skin, but they can involve virtually any lymph nodes or internal organs and poses a difficulty in diagnosis [2].

## Case presentation

A 24-year-old, sexually active homosexual male, presented with recurrent episodes of odynophagia, fever, enlarged tonsils, and oral thrush. Initial symptoms had started approximately seven months previously when the patient was advised to have an HIV serology performed. He was lost to follow-up during that period until the current presentation. Two weeks before admission he was diagnosed with HIV infection with a viral load of 190,087 copies/ml; the CD4 count was 232 cells/mcl and so started on antiretroviral therapy (dolutegravir/lamivudine/tenofovir) and Bactrim for pneumocystis prophylaxis.

During the current presentation, the patient reported high fever, odynophagia, sore throat, and weight loss of approximately 10 kg over the past six months. A physical examination revealed a fever of 38.5 degrees Celsius, blood pressure 120/70 mm Hg, heart rate 110/min, oxygen saturation (SpO2) 98% on room air, pale skin, anicteric sclera, poorly injected conjunctiva, oral thrush, and bilaterally enlarged tonsils, more prominent on the left side with some purple discoloration, as well as axillary and inguinal lymph nodes (LNs). The skin showed no abnormal lesions.

The workup showed the following: low hemoglobin level 9.2 g/dL, mean corpuscular volume 90 fl, white blood cells 5 200/mL (31.2% neutrophils, 58.8% lymphocytes), platelets 179 000/mL, and normal liver function tests. Cervical, chest, and abdominopelvic chest computed tomography (CT) showed the following: mediastinal and axillary LNs, heterogeneous multi-lobulated lesion partially compressing the pharynx, hepatosplenomegaly, and retroperitoneal, iliac, inguinal, and mesenteric LNs with peritoneal fluid. A tonsillectomy was performed, as well as an excisional LN biopsy in the right inguinal and the left axillary area, and the patient was discharged home pending the results.

**Figure 1:**
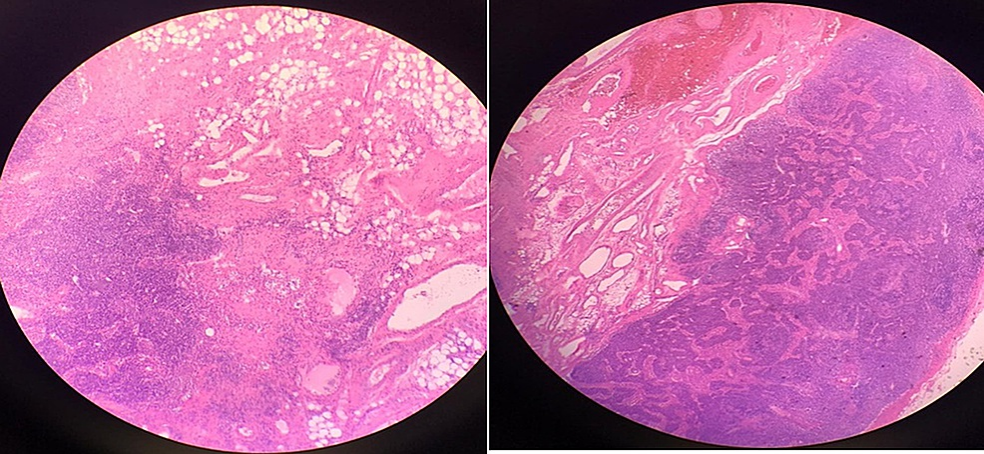
Histopathology image from the inguinal lymph nodes. Hematoxylin and eosin staining shows spindle cells in the lamina propria with extravasated red blood cells.

Preliminary results excluded mycobacterial infection, by a negative polymerase chain reaction (PCR) for mycobacterium, and excluded other infections by negative culture and gram stain and were in favor of a lymphoproliferative disorder, pending immunostaining. The patient was started on antiretroviral therapy (ART) (dolutegravir/lamivudine/tenofovir). Five days later, he started to complain of high fever, lethargy, and diffuse lower limb edema. At re-evaluation on day five, the patient looked ill and pale, with diffuse upper and lower limbs edema and a purple lesion at the right conjunctiva associated with eyelid edema, as well as new purple lesions in the retro pharynx and gingiva, and hypertrophy of a purple mass at the site of the tonsillectomy. The ART was stopped for possible immune reconstitution inflammatory syndrome (IRIS). The final pathology report of tonsils showed reactive lymphoid hyperplasia. Left axillary and right inguinal LN biopsies showed proliferation of small vessels associated with spindle cells involving the nodal parenchyma and the capsule diagnostic of nodal KS in a background of HIV lymphadenopathy (Figure 1).

**Figure 2:**
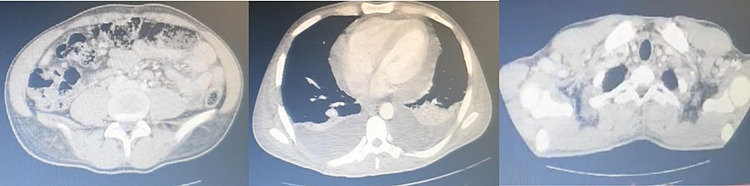
Non-contrast chest, abdomen, and pelvis CT showing bilateral pleural effusion and multiple lymph nodes.

The serology was negative for syphilis, Epstein-Barr virus, and hepatitis, whereas anti-cytomegalovirus (CMV) immunoglobulin (Ig)G was positive, so the patient started on ganciclovir. The patient reported diarrhea in which *Clostridium difficile* toxin A and B were negative. Fecal occult blood was positive, and stools showed a focal aggregate of inflammatory cells. A repeated chest, abdomen, and pelvis CT (Figure 2) was performed and showed a moderate bilateral pleural effusion, increased ascites, air-fluid level with distension of the intestine and colon stasis, hepatosplenomegaly, and retroperitoneal, iliac, inguinal, and mesenteric LNs.

*Mycobacterium tuberculosis* (MTB) PCR was negative. Urine, sputum, and blood cultures were taken and the patient was put on wide-spectrum antibiotics such as meropenem 1g intravenous every eight hours and vancomycin 1g intravenous every 12 hours along with antifungal therapy, and micafungin 100mg intravenous daily. After five days of presentation, all cultures were negative so superimposed infection was eliminated. There was no clinical improvement with increased anasarca and a persistent high-grade fever; a diagnosis of aggressive visceral KS was made. We reinitiated ART and administered the first dose of liposomal doxorubicin. The patient improved after one week, the lesions disappeared, and there was no more edema. The patient then developed pancytopenia after 15 days of presentation, with hemoglobin 5.3 g/dL, white blood cells 2,800/mL (32.0% neutrophils, 46.0% lymphocytes), and platelets 32,000/mL. A bone marrow biopsy showed hypercellularity (80%) with few interstitial reactive CD20+ B lymphocytes and numerous reactive CD3+ T lymphocytes, suggestive of an acute inflammatory process.

Serum taken for CMV showed a high viral load of 625 IU/ml, and the patient was started on therapeutic ganciclovir 5mg/kg intravenous every 12 hours. Vitamin B12 and folate were low at <150 pg/ml and 3.43 ng/ml, respectively; thus, cobalamin and folate were given. We administered a second dose of liposomal doxorubicin; however, the patient remained pancytopenic and continued to deteriorate with the appearance of pulmonary edema and pleural effusion, kidney failure, anemia, and coagulopathy. He died within three weeks of initiating highly active antiretroviral therapy (HAART). Until the death, no skin lesions were identified.

## Discussion

KS is an angioproliferative tumor of lymphatic endothelium-derived cells infected with HHV-8, typically occurring in patients with immunodeficiency. It has variable presentations that range from indolent, isolated dermatologic manifestations to fulminant disease with visceral involvement [1]. Although the skin and mucous membranes are the most frequently affected sites in KS [3], it may affect other parts of the body as well (e.g., lungs, stomach, intestine). The mucous membrane of the oral cavity is usually involved in KS of the head and neck, but it can involve the whole GI mucosa from mouth to anus. [4].

Although four KS variants have been described in the medical literature, all these forms likely represent different manifestations of the same pathological process, given that over 95% of KS lesions, regardless of their source or clinical subtype, have been found to be infected with HHV-8 [5].

Classic KS has a male predominance, with a male to female ratio of 17:1. It occurs primarily in the elderly of Eastern European and Mediterranean descent, who are at greater risk for secondary malignancies [6], and it typically presents with lesions confined to the lower extremities [6]. The second variant is the endemic or African form. This type is very aggressive, presents with local or generalized lymphadenopathy, and is found in South Africa in young children [7]. Cutaneous lesions are rare in this variant. The third variant is the transplant- or immunosuppression-associated form of KS. It typically begins between several months and several years after organ transplantation with immunosuppressive therapy. Skin lesions have been found in approximately half of the cases of this type; however, it can also affect the internal organs and LNs [8]. The fourth variant of KS is the AIDS-associated (epidemic) form. In the United States, it is the most common AIDS-associated tumor, and it occurs in approximately one-fourth of all patients with AIDS. In this type, KS affects men who have sex with men (MSM) 20 times more often than non-MSM patients with the same degree of immunodeficiency. This form is widely scattered, with no preferred locations, and involvement of the LNs and intestine occurs relatively early in the disease [9].

Visceral KS can appear in any organ, but it mainly involves the LNs, lungs, and gastrointestinal tract. Although 33-77% of patients with cutaneous KS have been found to have visceral lesions on autopsy, such lesions in the absence of skin manifestations are rare. The most frequent site involved in the gastrointestinal tract is the small intestine, followed by the colon and stomach [10]. Gastrointestinal KS is often without symptoms; however, it can present with nonspecific complaints such as nausea, vomiting, abdominal pain, and dyspepsia. More serious manifestations have also been reported, such as cases of hemorrhage, perforation, obstruction, diverticulitis, appendicitis, intussusception, and protein-losing enteropathy (PLE) [11].

The first description of PLE due to gastrointestinal KS in the medical literature was in 1987. The importance of investigating hypoalbuminemia in patients with AIDS was discussed in 1989 by Bouhnik et al [12], because severe cases associated with anasarca suggest small intestine pathology, given that it is possible this is the only sign of otherwise asymptomatic gastrointestinal KS.

In patients with HIV, diarrhea can have several etiologies. Gastrointestinal KS is highly associated with protein-losing enteropathy where fecal α1-antitrypsin >0.3 mg/g, wet stool have a sensitivity and specificity of 94% and 76%, respectively [11]. Therefore, PLE should be ruled out in any patient with AIDS who has hypoalbuminemia and gastrointestinal symptoms, especially when the loss of albumin is not explained by cardiac, renal, hepatic disease, or malnutrition. Moreover, PLE in patients with AIDS can be attributed to other etiologies such as lymphoma, tuberculosis, *Giardia*, *Cryptosporidium*, and CMV infection [13].

Oropharyngeal KS, particularly isolated KS of the tonsils, is rare with only a few cases reported in the literature. One of the earliest cases reports of KS located in the upper pole of the left tonsil was by Raikundalia in 1973, in a 38-year-old woman [14]. The oropharyngeal KS was treated surgically. Two KS cases of the tonsil in HIV-positive patients who underwent an excisional biopsy were described by Chetty and Batitang; however, both patients were lost to follow-up [15].

For a definitive diagnosis of KS, a biopsy or excision of the suspicious areas must be performed to look for the characteristic spindle cell proliferation in the dermis; these cells are currently believed to originate from lymphatic endothelium [16]. Immunohistochemistry shows expression of CD34, CD31, and CD2-40 [16]. Overall, visceral KS is typically associated with a poor prognosis.

The aim of treatment is usually palliative and primarily targets symptom improvement and prevention of progression. Regarding treatment options, therapy is started according to the severity of the disease on the initial presentation. If the disease is localized or minimally disseminated cutaneous KS, it usually responds to ART plus either cryotherapy, radiotherapy, or surgical resection of the lesion. ART causes resolution of KS lesions by suppressing HIV viral replication and increasing CD4 cell counts, without acting directly on HHV-8. If the disease is disseminated with visceral involvement, a combination of systemic chemotherapy with ART therapy is typically required [17]. Liposomal anthracyclines (e.g., doxorubicin) are considered as first-line systemic agents for the treatment of disseminated KS due to their favorable response rates and toxicity profiles [17].

In most prospective studies, patients with life-threatening or extensive KS received chemotherapy alone or adjuvant when the KS failed to respond to ART alone. The KS lesions of such patients regressed less often, and more slowly, than the lesions of patients with limited disease who received only ART. Chemotherapy could often be withdrawn when KS lesions regressed with the combination of ART and systemic treatment.

Although antiretroviral therapy is important for AIDS-associated KS improvement and resolution, in disseminated cases, a small proportion of patients exhibit deterioration in their condition following ART initiation. This condition is known as IRIS, and it is of varying severity and even fatality [18]. In the literature, KS-related IRIS (KS-IRIS) is rare and often not readily recognized compared with other more common diseases, such as tuberculosis, cryptococcal meningitis, *Mycobacterium avium*-*intracellulare* infection, and *Pneumocystis jirovecii* pneumonia.

A prospective study in Mozambique showed the presence of four independent factor predictors of KS-IRIS: hematocrit below 30%, KS pretreatment, high plasma viral HIV load ( ≥50 copies/ml), and detection of HHV-8 DNA in plasma [19]. Another study performed with the Chelsea and Westminster HIV cohort showed that KS-IRIS can be found in patients with high CD4 cell counts at the time of KS diagnosis in those treated mainly with nonnucleosides and protease inhibitors together, and also in those with KS-associated edema [20].

IRIS can be defined as a group of inflammatory disorders with progressive deterioration in clinical status after the recovery of the immune system, leading to worsening infections in patients infected with HIV after the initiation of ART [21]. KS-IRIS should have two or more of the following: increase in the number of KS lesions, exacerbations or appearance of lymphedema, lung opacities with an increase in CD4+ cell count ≥50 cells/mm3, and decrease of >1 log in viral load after starting ART [21].

In general, IRIS treatment is a challenge that depends on the clinical severity and disease agent because this entity is not well recognized and its appropriate management is still unclear. In moderate to severe IRIS cases, discontinuation of ART with corticosteroid use leads to decreased inflammation in varying degrees. However, the use of corticosteroids to treat KS-IRIS is not routinely indicated because it can decrease cellular immunity, permitting HHV-8 replication and tumor growth [21]. Thus, we stress the importance of early recognition of KS with the initiation of chemotherapy, and subsequently ART introduction.

One study showed that the use of chemotherapy in combination with continuing ART can lead to the resolution of KS-IRIS. This is highlighted in the brief report by Connick et al. in which the patient presented with facial KS and significant neck edema and lymphadenopathy while on HAART [22]. During chemotherapy (paclitaxel), the inflammation resolved coincidentally with a decrease in the CD4+ lymphocyte count. After a long period of ART and chemotherapy, this patient was finally cured. Thus, the authors concluded that clinicians should be aware of KS-IRIS from the beginning of the treatment. IRIS does not indicate failure of HAART or a need for changes in the antiretroviral regimen. Instead, adding chemotherapy to HAART can effectively control the symptoms of IRIS and resolve KS. In our case, the patient developed high-grade fever, generalized anasarca with the appearance of new lesions, and decreased hemoglobin within five days of ART. It was most likely a rapidly progressive aggressive presentation of visceral KS that did not respond to treatment.

## Conclusions

Visceral KS remains a severe disease. Primary therapy is liposomal doxorubicin with ART, and it is usually associated with a poor prognosis. It is now appreciated that HHV8 can cause several diseases, many of which had not been previously recognized. This case emphasizes the need to recognize and strongly consider visceral KS as a possible cause in any people living with HIV/AIDS with low CD4 counts. CT scan of the abdomen and pelvis can play an important role in the recognition of visceral lesions and lymph nodes and the diagnosis of KS. This case reflects the complexity of HIV and reinforces the need for greater awareness in screening regardless of whether a clear risk behavior has been identified.
